# Trust in the Leader and Trust in the Organization in Healthcare: A Concept Analysis Based on a Systematic Review

**DOI:** 10.1155/2024/8776286

**Published:** 2024-02-22

**Authors:** Venla Karikumpu, Arja Häggman-Laitila, Johanna Romppanen, Mari Kangasniemi, Anja Terkamo-Moisio

**Affiliations:** ^1^Department of Nursing Science, University of Eastern Finland, Kuopio Campus, P.O. Box 1627, Kuopio FI-70211, Finland; ^2^City of Helsinki Social Services, Health Care and Rescue Services Division, P.O. Box 6000, Helsinki FI-00099, Finland; ^3^Department of Nursing Science, University of Turku, Medisiina B, Turku FI-20014, Finland

## Abstract

*Orientation*. Trust is the central part of leadership and organizational culture and can often go unnoticed until it decreases. There is a lack of a comprehensive concept analysis of trust in the healthcare setting. *Research Purpose*. The research aim was to gather, assess, and synthesize previous empirical evidence from the field of healthcare about the concepts of trust in the leader and trust in the organization. *Motivation for the Study*. To create a comprehensive and generic concept analysis of trust in the leader and organization for the healthcare sector based on recent empirical studies. *Research Design and Method*. A concept analysis, which followed the method presented by Walker and Avant, was conducted as a systematic review that adhered to the PRISMA guidelines. A total of eight databases were searched for relevant literature and 42 articles were included. *Main Findings*. The definitions of trust in the leader and the organization were based on emotion and cognition. Trust in the leader emerged as a core feature of collaborative leader-employee relationships, whereas trust in the organization was a key construct of organizational functioning. Trust in the leader and the organization contributed to commitment, increased work production, enhanced collaboration, and improved workplace well-being. Defense mechanisms were identified as a new contrary concept, while justice was found to be a related concept. *Contribution*. Both trust in the leader and trust in the organization positively impact an organization, nurse leaders, and employees. Deeper knowledge of trust and its attributes will be critical to the operationalization and estimation of levels of trust in healthcare organizations. *Managerial Implications*. Trust in the leader and the organization can significantly influence the attractiveness of an organization, retention of personnel, productivity, and work-related well-being. Thus, this aspect should be measured and developed systematically while acknowledging the antecedents of trust building.

## 1. Introduction

Healthcare organizations constantly face strategic and operational changes due to various challenges, e.g., workforce shortages, inequalities in service coverage, and policy discrepancies [1]. At the same time, the general population is characterized by health inequalities, unmet health care needs, and demographic ageing [1, 2], factors which have considerably increased the amount of people with multiple health problems [3]. Responding adequately to these challenges requires healthcare reforms that involve innovative solutions concerning the provision of patient-centered, high-quality care [2]. A crucial part of this is ensuring that all individuals, regardless of social challenges or characteristics such as financial situation, place of residence, age or multimorbidity, have equal access to healthcare [2, 4].

Leaders in organizations are tasked with maintaining service continuity in various situations and supporting the workforce through changes in service provision [5]. As such, a comprehensive assessment of leadership competencies is a prerequisite for healthcare reforms [6]. Strong leadership competencies translate into decreased change resistance, which is inevitable during the implementation of reforms [7]. In addition to change leadership [8], along with visionary and encouraging leadership [9], leaders need to be well versed at developing and maintaining strong collaborative relationships with different stakeholders [6, 10, 11]. All of these aspects highlight the significance of trust in both the leadership and organization [12]. Trust is such an apparent part of leadership and the organizational culture that it often goes unnoticed until it decreases [13]. Thus, leadership should be periodically assessed, as well as measured prior to any large organizational changes as it has various positive effects [9, 13]; notably, increased cost-effectiveness [14], improved ethical competence [15], and work engagement among employees [16], along with enhanced overall quality of patient care [17].

The concept of trust is a topic of interest in several scientific disciplines, e.g., psychology, sociology, nursing, medicine [18], religion, philosophy, and business [19]. As such, it is unsurprising that trust is approached through several definitions and dimensions depending on the field of science [17, 19]. We identified three previous articles [17, 19, 20] that describe trust in the context of nursing. The oldest article, published in 2011, is a literature review that includes 20 studies published between the years 2002 and 2008 and limited to critical care. The results revealed that most of the published studies focused on trust within the nurse-patient relationship, with none of the articles describing relationships among healthcare staff [17]. Another article, published in 2014, presented the results of a concept analysis. The study material included interviews with 28 nurses and 11 nurse managers working in acute and community care. However, the analysis focused solely on the antecedents, attributes, and consequences of trust, while various definitions and related concepts were not considered [20]. Most recently, trust was approached by using concepts from other disciplines, e.g., arts or business, by employing Watson's theoretical perspective. The provided examples centered around the nurse-patient relationship as a helping-trusting relation, while the relationships between leaders and nurses were not assessed [19]. Thus, there is a limited amount of studies on trust from the healthcare setting. Furthermore, the studies that do exist are based on historical data and do not provide a comprehensive view of the concept of trust for the healthcare sector. This highlights the need for a comprehensive concept analysis on the issue of trust within healthcare. Furthermore, the nurse-patient relationship starkly differs from the nurse-nurse leader relationship, as well as the relationship between a nurse and the organization, due to imbalances in hierarchical power. Trust can be regarded as a dynamic social construct that changes over time [19]; this further proves that the empirical literature on trust in the healthcare sector needs to be updated. The present article fills this research gap by presenting a concept analysis of trust that is based on the systematic review of empirical data and performed according to the method introduced by Walker and Avant [21]. The present review identified empirical studies that described trust in leaders and/or healthcare organizations with the underlying goal of developing an instrument for future studies. Even when leadership and the organization are closely intertwined, essential differences exist between them; thus, the contents and features of these two concepts have been differentiated.

The aim of this study was to gather, assess, and synthesize previous empirical evidence from the field of healthcare about the contents and characteristics surrounding the concepts of trust in the leader and trust in the organization. The research was performed under the guidance of the following questions:

1. How are the concepts of trust in the leader and trust in the organization described and defined and what kind of attributes have been associated with these concepts in empirical research?
2. Which antecedents and consequences of trust in the leader and trust in the organization have previously been identified?
3. What kinds of borderline, related, and contrary cases of trust in the leader and trust in the organization have been identified?

## 2. Materials and Methods

### 2.1. Design

A concept analysis based on a systematic review was conducted according to the PRISMA checklist [22] (Supplementary table 1) and the method presented by Walker and Avant [21].

### 2.2. Search Methods

A systematic literature search was conducted in March 2021 and updated in January 2024; the search was performed across the following eight databases: Medic; PsycINFO; MEDLINE (Ovid); SocIndex; PubMed; CINAHL; Web of Science; and Scopus. The search strategy was created in collaboration with an information specialist. The search terms (Table 1) covered trust-related terms in both Finnish and English that were combined by using Boolean operators. The inclusion and exclusion criteria are presented in Table 1, and the searches were limited to peer-reviewed articles published between March 2010 and December 2023 in Finnish or English.

**Figure 1:**
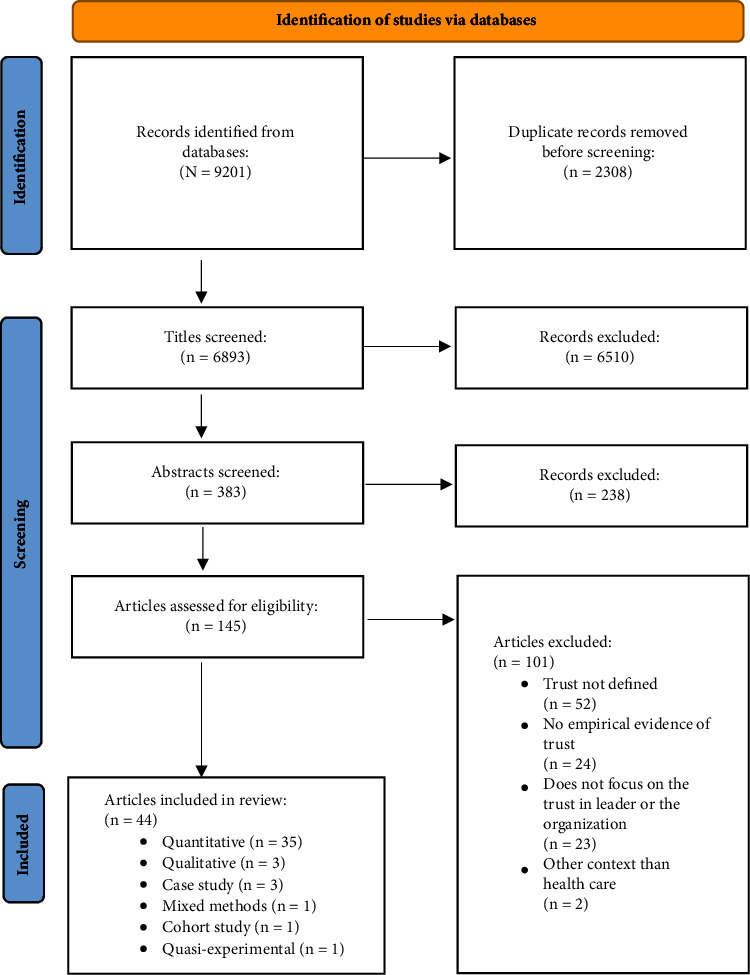
PRISMA flowchart of search results [22].

The search yielded a total of 9,201 titles that were moved to RefWorks, after which the results of the updated search were transferred into Covidence. Following duplicate removal (*n* = 2,308), a total of 6,893 titles were independently screened by two researchers (VK and JR). Next, the abstracts (*n* = 383) of relevant articles were independently screened by two researchers (VK and JR), after which the results were compared. Consensus was achieved by discussion, and a third researcher (AT-M) was consulted when a consensus could not be reached. A total of 145 full-text articles were assessed for eligibility. The final data include 44 original articles (Figure 1). The results of the selection process were discussed with two other researchers (AT-M and AH-L), who provided an additional assessment of the relevance of chosen articles [23].

### 2.3. Quality Appraisal

The quality of all of the included studies was independently evaluated by two researchers (VK and JR) according to a checklist from the Center for Evidence-Based Management [24] (Supplementary table 2). The cutoff point for acceptance was set at 50% of the possible points [25].

A cross-sectional checklist (12 items) was used to appraise the quality of quantitative studies. The strengths of these studies included a clear research frame, solid methods, application of reliable instruments, and a clear presentation of the results. However, confounding factors were sparsely presented, which was assessed as a weakness. Articles which reported case studies, along with one mixed methods study, were assessed by the case study checklist (10 items). The strengths of these studies were clear descriptions of settings, data collection, and credible outcomes, whereas unclear descriptions of the researchers' roles and analytical methods emerged as weaknesses. The qualitative checklist (10 items) was employed to appraise the quality of interview studies. The strengths of these studies included clear descriptions of study design, context, fieldwork, and outcomes. The most common weakness of the included qualitative studies was insufficient description of researchers' contributions and roles. The quality of the quasiexperimental study included in this review was appraised using the checklist of a controlled study (12 items); the checklist of a cohort or panel study (12 items) was employed to assess the quality of the one identified cohort study. The strengths of these studies included strong methods and sufficient sample size as was the case in quantitative studies, and missing descriptions of confounding factors were assessed as the primary weakness.

Following the independent assessment of article quality, the two researchers (VK and JR) discussed their findings and consulted a third researcher (AT-M) in the case of any disputes. As a result of the quality assessment, two articles [26, 27] were excluded from the review due to a low level (<50%) of accrued points [25]. Thus, the review included a total of 42 articles.

### 2.4. Data Extraction, Concept Analysis, and Degree of Evidence

A matrix for the data extraction was developed for the purpose of this study. It included information about study design, aim, context, data collection, analytical method, and results concerning the concepts of trust. In the first phase of the analysis, the data were repeatedly assessed to identify any descriptions of trust in the leader and trust in the organization; this approach was in line with the concept analysis method presented by Walker and Avant [21]. The original expressions related to the research questions were condensed and then moved into the results section of the matrix. Next, condensed expressions of the definitions of trust in the leader and trust in the organization were compared according to their similarities and differences and then divided into three groups based on content. The condensed expressions concerning the attributes (Table 2) and antecedents (Table 3), along with borderline, related, and contrary cases as well as consequences (Table 4), of trust were then categorized into the following two groups: trust in the leader and trust in the organization. In the next step, the expressions were compared according to similarities and differences to identify sub, upper, and main groups. The identified groups were named according to their contents [69]. Lastly, the degree of evidence was evaluated to identify the essential main outcomes [70]. The strongest degree of evidence was indicated with A, consisting of meta-analyses, systematic reviews, and randomized control trials (RCTs). Degree B included cross-sectional studies, whereas C indicated studies with qualitative study design. The lowest degree of evidence was indicated with D, including observational designs. In addition, the study limitations, quality of results, and directness of evidence across different studies influenced the conclusion of the degree of evidence [70].

## 3. Results

### 3.1. Study Characteristics

The data consisted of quantitative surveys (*n* = 33), qualitative interview studies (*n* = 3), and case studies (*n* = 3). In addition, one mixed methods study, one cohort study, and one quasiexperimental study were included in the data (Figure 1). All of the included articles were published between 2010 and 2023, with most (*n* = 32) published after the year 2015. The studies were conducted in Turkey (*n* = 9), the USA (*n* = 7), Australia (*n* = 3), South Africa (*n* = 3), Canada (*n* = 3), Finland (*n* = 2), Norway (*n* = 2), Pakistan (*n* = 2), Spain (*n* = 2), Sweden (*n* = 2), Denmark (*n* = 1), Iran (*n* = 1), Israel (*n* = 1), Korea (*n* = 1), and Nigeria (*n* = 1). Moreover, two articles reported the results of a study that had been conducted in two countries, USA and England, as well as Finland and Norway (Supplementary table 3).

Of the identified studies, 24 focused on trust in the leader and 18 concerned on trust in the organization. The studies were mainly conducted in the context of public healthcare (*n* = 23), while one study covered the private healthcare sector; three studies reported results concerning both the public and private healthcare sectors. It should be noted that 14 studies mentioned the healthcare context but did not clearly define it. In one of the included studies [62], the healthcare employees represented only 10% of the participants. A total of 29 articles reported employees' perspectives of trust, whereas four studies covered the leaders' perspective; both the perspectives of employees and leaders were described in nine articles (Supplementary table 3). Finally, the reference lists of the included studies were searched to determine the contents of the provided definitions. As a result, the definitions in the next section are based on original articles that were published outside of the time range included in the present review. The connection to the current data is presented in Supplementary table 4.

### 3.2. Definitions of Trust in the Leader

Trust in the leader was defined in 11 different ways, which could be divided into emotion-based and cognition-based trust. The emotion-based definitions described trust as the trustor's willingness to be vulnerable in a situation where they are performing an action which is important to them [71, 72]. In such situations, the leader or employee ventures into a position of interdependence [73], i.e., the trustor takes a risk of trusting the trustee without the ability to control their actions [71, 74, 75]. The emotion-based definitions of trust included reciprocal positive expectations of benevolence [71, 72, 74–77] in accordance with open interaction [73, 78]. The cognition-based definitions of trust were related to belief in the competence of the trustee [78, 79].

### 3.3. Definitions of Trust in the Organization

Trust in the organization was defined in 12 different ways, which could be divided into emotion based, cognition based, and predictability of the organization's functions. The emotion-based definitions included a trustor's willingness to be vulnerable when a group or organization performs an action that was important to the trustor [80–82]. Therefore, these definitions included trustees' positive feelings about organizational support and confidentiality [71, 83, 84]. The cognition-based definitions described the reciprocal expectation of competence and reliability [71] to promote mutual interests even in situations where the trustee could act based on self-interest [84, 85]. Trust in the predictability of the organization's functions was described as the integrity [86–88] and ethicality [89] of an organization's functions. In addition, trust was described as the trans-sectional presence of reciprocal trust across different organizational levels [81, 90].

### 3.4. Attributes of Trust in the Leader

The attributes of trust in the leader could be described as the core collaborative leader-employee relationship, positive expectations of benevolence, social interaction, a leader's competence, and risk and vulnerability (Table 2). Descriptions of the core collaborative leader-employee relationship stated that trust is a critical foundation of all actions. Thus, it is recognized as a sensitive, reciprocal process that requires time to develop. Furthermore, it is considered a fragile construct that can be easily broken. The attribute of positive expectations of benevolence was described as the trustor's convictions about the trustee's motives, intentions, and actions. In other words, benevolence means that a trustee's ethical and moral actions are not based on self-interest. Moreover, a trustee's strategic actions are expected to lead to reciprocal benefits (Table 2).

According to the included articles, social interaction is realized as reciprocal and positive communication between the trustor and the trustee, whereas a leader's competence was described as the ability to lead, create a sense of security, and inspire employees through an expert role. As such, a leader's competence enables employees to demonstrate their professionalism and focus on their duties. The attribute of risk is based on the trustor's expectations that the trustee's actions or intentions cannot be controlled. Furthermore, trust creates a situation in which both parties (trustor and trustee) are willing to be exposed to reciprocal vulnerability (Table 2).

### 3.5. Attributes of Trust in the Organization

The identified articles included the following attributes of trust in the organization: a key construct of organizational functions; positive expectations of benevolence; and social interaction (Table 2). Trust, as a key construct of organizational functions, is an important tool in leading collaborative relationships and developing professionalism. It is also crucial for success across various organizational levels. The attribute of positive expectations of benevolence is related to situations in which employees trust that the organization will take care of employee well-being and problems. The attribute of positive expectations of benevolence describes how an employee functions within an organization, which includes interactions with the leader. Social interaction also included a leader's willingness to act honestly and predictably (Table 2).

### 3.6. Antecedents of Trust in the Leader

The antecedents of trust in a leader were leadership skills, consistent action, open interaction, and collaboration (Table 3). The leadership style, which falls under leadership skills, favors collaborative relationships that advance trust, i.e., transformational, ethical, and authentic leadership. Trust could also be promoted by the appropriate use of different forms of power, namely, reward, legitimate, and referent power. Therefore, leadership skills were described through the readiness to modify leadership practices, for example, through actively informing and involving employees (Table 3).

Consistent action included a leader's benevolence, which includes the ability to identify employees' feelings. This attribute was described through a leader's ability to acknowledge the diverse and individual perspective of their employees. Furthermore, this attribute included the competent actions of consistent leaders, for example, ability to make decisions and motivate employees. A leader's presence manifests as authenticity, visibility, and integration into the work community, all of which support trust formation. Lastly, a leader's involvement, including employee encouragement, as well as employees' impact on planning processes or decision-making, was identified as antecedents of trust in terms of a leader's consistent action (Table 3).

Open interaction included knowledge sharing and the openness of the work community. Reciprocally, active listening, along with the provision of feedback and experiencing a connection through profound communication, were important to building trust and could be strengthened by a leader's positive attitude. In addition, collaboration consisted of commitment by both the leaders and employees, as well as reciprocal collectivity. Trust is built when there is responsibility in collaboration, i.e., each member of the team takes care of their own duties. Collaboration also includes employees' positive feelings about organizational actions, or—in other words—their ability to thrive at work (Table 3).

### 3.7. Antecedents of Trust in the Organization

According to the identified articles, the antecedents of trust in the organization are leadership skills and the role of employees (Table 3). Leadership skills, when considered in the context of the organization, include approaches such as authentic and transformational leadership. This can be extended to certain positive characteristics of an organization, such as stability, competence, honesty, and loyalty. The role of employees consists of employees' openness in sharing tacit knowledge. Therefore, this aspect was described through concepts such as work engagement, job satisfaction, and obsession with work performance. Moreover, this aspect includes employees receiving fair treatment and being committed to the organization both affectively and normatively (Table 3).

### 3.8. Consequences of Trust in the Leader and Trust in the Organization

The consequences of trust in the leader and the organization consisted of commitment, increased productivity at work, increased collaboration, and increased workplace well-being (Table 4). When considered from the lens of trust in the leader, commitment to work was described as the commitment of the work community to the constant development of activities. As commitment is a reciprocal process, it can be categorized as affective, normative, and continuance organizational commitment. When shifting to trust in the organization, commitment was categorized into work and the organization. Additional definitions of organizational commitment stated that trust increases both continuance and affective commitment (Table 4).

When considering trust in the leader, increased productivity at work included better motivation and creativity to work, both of which increase innovativeness. For instance, work performance improved when a team or an individual successfully accomplished their work duties. In addition, trust in the leader increased leaders' and employees' experiences of efficiency. Furthermore, trust improved employee attitudes, which decreased cynicism towards changes. From the lens of trust in the organization, increased productivity at work included experiences of high-quality care and a concern for safe behavior. Furthermore, trust was found to increase the performance of a team, as well as organizational efficiency, productivity, and the conscientiousness of employees (Table 4).

In terms of trust in the leader, increased collaboration consisted of common goal orientation and shared decision-making. For instance, polite interactions between team members increased as employees felt encouraged to communicate with the leader, even about sensitive and/or confidential issues. Concerning trust in the organization, increased collaboration comprised good communication, cohesion, and an employee's ability to identify with the leader's values; the published research suggested that this makes clinical decision-making easier. Furthermore, this aspect of trust was described as an ethical atmosphere in which employees target common goals, are polite, and demonstrate selflessness (Table 4).

When considering trust in the leader, increased workplace well-being manifested as improved work satisfaction, especially among employees. Trust in the leader facilitates improvements in satisfaction with change processes and the organization. Furthermore, this aspect describes mental and physical health, which translates to safe overall behavior. From the lens of trust in the organization, increased workplace well-being improved job satisfaction among employees. Moreover, this trust enables employees to better manage their stress and perform high-quality work (Table 4).

### 3.9. Overview of the Main Results of the Concept Analysis

**Figure 2:**
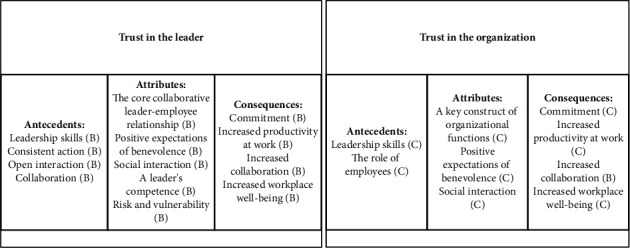
Antecedents, attributes, and consequences of trust in the leader and trust in the organization.

The antecedents, attributes, and consequences of trust in the leader and trust in the organization are presented in Figure 2, with the level of evidence specified for each finding. The findings for trust in the organization showed lower levels of evidence when compared to findings for trust in the leader.

### 3.10. Borderline, Related, and Contrary Concepts of Trust in the Leader and Trust in the Organization

The borderline, related, and contrary concepts of trust in the leader and the organization included the same concepts, with the exception of defense mechanisms, which were a part of the contrary concept of trust in the leader. The psychological contract emerged as a borderline concept of trust because both concepts contained several joint attributes. At the same time, it was also seen as an antecedent of trust, as the psychological contract is based on reciprocal expectations of actions and keeping the promises of the trustee [44, 66]. Justice was identified to be a related concept of trust [52] as it is also part of the core of a healthy organization [59]. Furthermore, the concept of justice is embedded in the interactions that produce social welfare, as is trust [62]. Thus, interactional and distributive justice depends on the quality of the collaborative relationship when integrated into trust in the leader [60].

Distrust was recognized as a contrary concept to trust [34], with a lack of open communication and employees' perceptions of injustice exerting a negative influence on trust [31, 91]. Employees' experiences of betrayed promises, the inability to get involved in decision-making, and the lack of a leader's support enhance distrust [91]. Furthermore, defense mechanisms [46] emerged as a contrary concept of trust and described as the actions or thoughts that protect individuals, groups, or organizations when confronting unpleasant realities [48]. These unpleasant realities were described through decreased trust—mainly due to insufficient collaboration—and a lack of communication, both of which lead to situations in which the trustor is not willing to be vulnerable towards the trustee [48].

### 3.11. Model Case of Trust in the Leader and Trust in the Organization

A healthcare organization has recruited a nurse who will work in the ward. Another colleague introduces this nurse to the work. The colleague says, “Welcome to our ward and to our hospital. Here, we have warm, collaborative relationships with colleagues and especially with our leader (attributes: a key construct of organizational functions and a core of collaborative leader-employee relationship). I have learned to trust her because she always stands up for us and treats us fairly (attribute: positive expectations of benevolence). It is easy to talk to her, even about difficult things and feelings. She listens, seriously considers the issues we talk about, but also asks for our opinion (attribute: reciprocal social interaction). Our leader has a Master's degree (attribute: competence), but she is always willing to improve as a leader. She constantly asks us for feedback and wants to know what aspects she should develop further (attribute: vulnerability). Because of this, our leader enables us to face our deficits, grow in our profession, and in that sense accept the risk of trusting each other (attribute: risk).”

The nurse continued, “This all is possible because the hospital invests in every level of the organization including employee well-being and keeps promises concerning professional development and employee involvement in decision-making (attribute: positive expectations of benevolence). The hospital strategy includes the following key values of leadership: open, honest, and predictable interactions (attribute: social interaction). Therefore, it is easy to expect that your leader will also behave in this way (antecedents: consistent action and open interaction). The hospital constantly educates leaders (antecedent: leadership skills) and develops forums for employee involvement (antecedents: the role of employees and collaboration).”

The nurse concluded, “Nurse turnover in the hospital and in our ward is the lowest in this city area (consequence: commitment). Our peers are innovative and work together efficiently (consequences: increased productivity at work and increased collaboration), and everyone demonstrates a positive attitude towards change without resistance (contrary concept: defense mechanisms). We do not have many absences from work (consequence: increased workplace well-being) and our hospital has been judged to be a fair workplace (related concept: justice). I appreciate this hospital a lot, as I have negative experiences with trust from previous workplaces. For example, one of my previous leaders had favorite employees and shared information with them that was withheld from the rest of us (contrary concept: distrust). We hope that you also share these values and can commit to the hospital (borderline concept: psychological contract).”

## 4. Discussion

To the best of our knowledge, this research represents the first concept analysis that is based on a systematic review of empirical studies and describes the concepts of trust in the leader and trust in the organization in the healthcare context. Previous literature is characterized by limited descriptions of the concept of trust, most of which focus on either the nurse-patient relationship or a special context within healthcare; furthermore, other descriptions of trust are based on a single interview study [17, 19, 20]. The present study provides a comprehensive analysis of the concept that can be applied to the context of public healthcare. This concept analysis enhances the prevailing nursing literature by describing the definitions and characteristics of trust based on a systematic review of international literature. Furthermore, the topicality of the data in this study (published between 2010 and 2023) provides important insight into the current state and dynamic nature of trust between the leader, employee, and organization. Recent developments within healthcare, such as remote leadership, have caused trust to become recognized as one of the core factors of successful collaborative relationships [92].

The presented descriptions of trust in the leader emphasize the leader's role, whereas the identified components of trust in the organization mostly focus on the functioning of an organization. Trust in the leader was found to include more interactional elements, e.g., risk and vulnerability, than trust in the organization. Moreover, open interactions and collaboration were identified as antecedents of trust in the leader; this was not the case for trust in the organization. The similarities between the identified consequences of trust in the leader and trust in the organization mean that future research should focus on this content. Emotion- and cognition-based definitions were used to describe both trust in the leader and trust in the organization. Trust in the leader was found to be at the core of the leader-employee collaborative relationship and included positive expectations of benevolence, social interaction, the leader's competence, as well as risk and vulnerability. Trust in the organization was identified as a key construct of organizational functioning and also included positive expectations of benevolence and social interaction.

Based on the literature assessed in this review, trust in the organization has only received a limited amount of research attention, with most of the presented findings usually characterized by weak evidence. Also, the borderline, related, and contrary concepts of trust in the leader and trust in the organization have only been covered in a handful of studies. The results concerning trust in the leader demonstrated the strongest overall level of evidence. The content analysis revealed that the definitions of trust could be divided into emotion- and cognition-based concepts, along with the predictability of organizational functioning. Nevertheless, this division was not possible for the attributes, antecedents, and consequences of trust in the leader and trust in the organization; thus, the concept of trust must be further examined in empirical healthcare environments. These results would be important to the eventual formulation of an instrument which could divide trust in healthcare organizations into leader- and organization-centric factors. It is important to note that the present review did not address trust in the client and trust in the coworker, which have also been recognized as important aspects in overall trust. Moreover, it has been found that the levels of trust in healthcare organizations, especially emotion-based trust, are rather low [93, 94]. As such, the presented results could be relevant to the development of healthcare as emotion-based trust was found to be firmly linked with the leader.

The presented results, which reflect empirical research, support the theoretical definitions of trust described in different disciplines of science [71, 78, 90]. Trust serves as one of the core pillars of an organization, with collaborative relationships—which include positive expectations of benevolence and interaction—typical of environments with high levels of trust. The presented results agree with what has been reported in previous concept analyses; nevertheless, there are also notable differences between the present review and prior research. For instance, Mullarkey and colleagues [17], along with McCabe and Sambrook [20], highlight the integrity, openness, and competence of a leader when discussing trust. Furthermore, Mullarkey and colleagues [17] emphasize that trust is a central driver of organizational success, expectations of behavior, as well as the presence of risk and dependence. In this study, risk and vulnerability, which involve a leader's competence, were linked with trust in the leader based on the analyzed studies. The results indicate that it is important to focus on these attributes to build trust both in the leader and in the organization.

The finding that leadership skills and the role of employees are antecedents of trust was supported by previous literature [17, 20, 95]. Destructive leadership was not extensively discussed in the included articles. This is logical because the research into this leadership style is sparse [96] and should be considered in further studies. There is some evidence from other disciplines that a leader's destructive behavior, for example, exercising coercion, invisibility, or impoliteness, will decrease trust [95, 97]. The identified antecedents highlight aspects which should hold a central role in organizational reforms that strive to achieve trust. For instance, reducing—and even possibly eliminating—destructive leadership is the key to building trust in the leader.

According to the research included in this review, integrity and workplace well-being are strong and clear consequences of trust in the leader and trust in the organization, a finding which mirrors previous concept analyses of trust [17, 20, 95]. As has been stated previously, trust positively affects individual well-being, the productivity of a team and its leader, as well as the quality of patient care [17, 20]. The finding that commitment is a consequence of trust is also supported by previous concept analyses [17, 95]. These consequences reinforce the importance of developing trust in the leader and in the organization.

### 4.1. Strengths and Limitations

The present study was influenced by several strengths and limitations. The primary strength of this study was that an extensive amount of data was collected from empirical research conducted in the healthcare context; these data provided broad answers to the research questions. Furthermore, the identified original articles mostly demonstrated sufficient quality, and the potential biases were acknowledged and described. More specifically, the identified original articles applied relevant methodologies and included adequate samples [98].

The level of evidence underlying the results presented in empirical studies included in this review was low due to the lack of randomized controlled trials; this can be regarded as a limitation of this study. Furthermore, determining valid, empirical reference definitions for trust was outside of the scope of this paper because it requires a qualitative approach; as such, this remains a challenge for future research. Nevertheless, the empirical example of a model case was included to strengthen the reliability of the presented results, according to the concept analysis method of Walker and Avant [21]. The fact that only one researcher performed the database searches decreased the trustworthiness of the research. Nevertheless, the other researchers supported the research process by assisting in searching for relevant knowledge and critically reviewing the formed concepts, with progression to the next study phase requiring a consensus. Furthermore, the conscious application of a negative term (not patient and client) in the Boolean operators might have removed some relevant outcomes from the search. Similarly, the decision to avoid gray literature could be considered a limitation [98].

## 5. Conclusions

The current review illustrates that there is a lack of concept analyses regarding trust in the leader and in the organization for the field of healthcare; there is a clear need for further empirical research into this phenomenon. Furthermore, the dimensions, definitions, attributes, antecedents, consequences, and other concepts of trust presented in this study need further clarification. There is far less data on trust in the organization, while the low level of evidence, determined via a clear methodology, necessitates further research. Furthermore, there is a need for an instrument that includes comprehensive descriptions of both concepts of trust, i.e., trust in the organization and trust in the leader. The presented research is relevant because deep knowledge of trust and its attributes will be critical to the operationalization and estimation of levels of trust in healthcare organizations. Furthermore, the concept of trust is clarified when justice was identified as a new related concept to trust, while defense mechanisms represented a contrary concept. Hence, the presented findings could be used to develop interventions for building both trust in the leader and trust in the organization. It would be important to apply methodologies that can determine the organizational costs and effectiveness of these types of interventions.

## 6. Implications to Nursing Management

Trust in the leader and in the organization exerts significant consequences on the attractiveness of a certain organization, retention of personnel, productivity, and work-related well-being. Thus, it should be measured and developed systematically in a way that acknowledges the antecedents of trust building. The results of this concept analysis may be utilized in the development of such an instrument. Building and maintaining trust are strongly associated with open and honest communication and reciprocal interactions; these are aspects to which nurse leaders should focus on in their daily work. Organizations should support leadership practices that place interaction in the center of the concept, as this would be beneficial to both trust development and a psychologically safe organizational culture. More mentoring and education are needed in this regard. Furthermore, educational organizations may take the current results into account by planning and developing their curricula. In addition, the development of interventions that primarily aim to build and maintain trust, for instance, by preventing distrust and defense mechanisms, may be beneficial for healthcare organizations.

## Figures and Tables

**Table 1:** Search strategy.

Databases	Search terms	Inclusion criteria	Exclusion criteria
Medic	luottamu^*∗*^ AND terveydenhuol^*∗*^	(i) Trust in the leader or in the organization (ii) Context of healthcare (iii) Empirical study designs	(i) Trust not defined (ii) No empirical evidence of trust (iii) Trust outside the leader or the organization (iv) Other context than healthcare (v) Reviews or meta-analyses (vi) Low quality of articles
PubMed	trust^*∗*^ AND “healthcare” AND (leadership OR management) AND NOT (patient^*∗*^ OR client^*∗*^)		
Scopus			
Web of Science			
CINAHL			
PsycINFO			
SocINDEX			
MEDLINE (Ovid)			

**Table 2:** Attributes of trust in the leader and trust in the organization with references and level of evidence.

Attributes	Trust in the leader	Trust in the organization
The core collaborative leader-employee relationship	[28–34] Level of evidence: B	
A key construct of organizational functions		[35–43] Level of evidence: C
Positive expectations of benevolence	[29, 30, 33, 34, 44–49] Level of evidence: B	[40, 41, 43, 50–52] Level of evidence: C
Social interaction	[29, 44, 48, 49, 53, 54] Level of evidence: B	[40, 43, 52, 55] Level of evidence: C
A leader's competence	[34, 44, 45, 49] Level of evidence: B	
Risk and vulnerability	[30, 33, 34, 45, 48, 56, 57] Level of evidence: B	

**Table 3:** Antecedents of trust in the leader and trust in the organization with references and level of evidence.

Antecedents	Trust in the leader	Trust in the organization
Leadership skills	[30, 32, 44, 47, 49, 58, 59] Level of evidence: B	[36–38, 50, 55] Level of evidence: C
Consistent action	[28, 34, 44, 45, 48, 54, 59, 60] Level of evidence: B	
The role of employees		[37, 40, 52, 61] Level of evidence: C
Open interaction	[34, 44, 56, 60] Level of evidence: B	
Collaboration	[32, 34, 44, 48, 62] Level of evidence: B	

**Table 4:** Consequences of trust in the leader and trust in the organization with references and level of evidence.

Consequences	Trust in the leader	Trust in the organization
Commitment	[39, 40, 43, 44, 53, 54, 59] Level of evidence: B	[37, 40–43, 50, 63] Level of evidence: C
Increased productivity at work	[29, 30, 32, 44, 46, 48, 56, 58, 62, 64, 65] Level of evidence: B	[35, 36, 38, 42, 55, 66] Level of evidence: C
Increased collaboration	[44, 49, 57, 64, 67, 68] Level of evidence: B	[35, 42, 51, 61] Level of evidence: B
Increased workplace well-being	[29, 39, 44, 47, 65] Level of evidence: B	[41, 42] Level of evidence: C

## Data Availability

The data used to support the findings of this study are from previously reported studies, which have been cited.

## Supplementary Materials

Supplementary Materials Supplementary Materials include tables supplementing the research article. There is further information about the Materials and Methods, providing the PRISMA checklist of the review process (Supplementary table 1) and the consensus of quality appraisal (Supplementary table 2). In addition, adhering to the Data section, there are summarized details about all reviewed articles (Supplementary table 3) and have tabulated the definitions of trust including in original articles (Supplementary table 4).
