# Supplementary material for: Trust in the Leader and Trust in the Organization in Healthcare: A Concept Analysis Based on a Systematic Review
Source: J Nurs Manag. 2024 Feb 22;2024:8776286. doi: 10.1155/2024/8776286 (PMC11918882; doi:10.1155/2024/8776286)
Supplement: Supplementary Materials — include tables supplementing the research article. There is further information about the Materials and Methods, providing the PRISMA checklist of the review process (Supplementary table 1) and the consensus of quality appraisal (Supplementary table 2). In addition, adhering to the Data section, there are summarized details about all reviewed articles (Supplementary table 3) and have tabulated the definitions of trust including in original articles (Supplementary table 4). [file 8776286.f1.docx]

**SUPPLEMENTAL FILES**

**Supplementary table 1. PRISMA checklist of review process (Moher et al. 2009).**

| **Section and Topic** | **Item #** | **Checklist item** | **Location where item is reported** |
| --- | --- | --- | --- |
| **TITLE** | | |  |
| Title | 1 | Identify the report as a systematic review. | p. 1 |
| **ABSTRACT** | | |  |
| Abstract | 2 | See the PRISMA 2020 for Abstracts checklist. | n/a |
| **INTRODUCTION** | | |  |
| Rationale | 3 | Describe the rationale for the review in the context of existing knowledge. | pp. 2-3 |
| Objectives | 4 | Provide an explicit statement of the objective(s) or question(s) the review addresses. | p. 3 |
| **METHODS** | | |  |
| Eligibility criteria | 5 | Specify the inclusion and exclusion criteria for the review and how studies were grouped for the syntheses. | p. 4 |
| Information sources | 6 | Specify all databases, registers, websites, organisations, reference lists and other sources searched or consulted to identify studies. Specify the date when each source was last searched or consulted. | p. 3 |
| Search strategy | 7 | Present the full search strategies for all databases, registers and websites, including any filters and limits used. | pp. 3-4 |
| Selection process | 8 | Specify the methods used to decide whether a study met the inclusion criteria of the review, including how many reviewers screened each record and each report retrieved, whether they worked independently, and if applicable, details of automation tools used in the process. | p. 4 |
| Data collection process | 9 | Specify the methods used to collect data from reports, including how many reviewers collected data from each report, whether they worked independently, any processes for obtaining or confirming data from study investigators, and if applicable, details of automation tools used in the process. | pp. 3-4 |
| Data items | 10a | List and define all outcomes for which data were sought. Specify whether all results that were compatible with each outcome domain in each study were sought (e.g. for all measures, time points, analyses), and if not, the methods used to decide which results to collect. | pp. 3-4 |
|  | 10b | List and define all other variables for which data were sought (e.g. participant and intervention characteristics, funding sources). Describe any assumptions made about any missing or unclear information. | pp. 3-4 |
| Study risk of bias assessment | 11 | Specify the methods used to assess risk of bias in the included studies, including details of the tool(s) used, how many reviewers assessed each study and whether they worked independently, and if applicable, details of automation tools used in the process. | pp. 3-6 |
| Effect measures | 12 | Specify for each outcome the effect measure(s) (e.g. risk ratio, mean difference) used in the synthesis or presentation of results. | Tables 2-4 |
| Synthesis methods | 13a | Describe the processes used to decide which studies were eligible for each synthesis (e.g. tabulating the study intervention characteristics and comparing against the planned groups for each synthesis (item #5)). | pp. 5-6 |
|  | 13b | Describe any methods required to prepare the data for presentation or synthesis, such as handling of missing summary statistics, or data conversions. | n/a |
|  | 13c | Describe any methods used to tabulate or visually display results of individual studies and syntheses. | Tables 2-4, Figure 2 |
|  | 13d | Describe any methods used to synthesize results and provide a rationale for the choice(s). If meta-analysis was performed, describe the model(s), method(s) to identify the presence and extent of statistical heterogeneity, and software package(s) used. | n/a |
|  | 13e | Describe any methods used to explore possible causes of heterogeneity among study results (e.g. subgroup analysis, meta-regression). | n/a |
|  | 13f | Describe any sensitivity analyses conducted to assess robustness of the synthesized results. | n/a |
| Reporting bias assessment | 14 | Describe any methods used to assess risk of bias due to missing results in a synthesis (arising from reporting biases). | p. 14 |
| Certainty assessment | 15 | Describe any methods used to assess certainty (or confidence) in the body of evidence for an outcome. | p. 6 |
| **RESULTS** | | |  |
| Study selection | 16a | Describe the results of the search and selection process, from the number of records identified in the search to the number of studies included in the review, ideally using a flow diagram. | Figure 1 |
|  | 16b | Cite studies that might appear to meet the inclusion criteria, but which were excluded, and explain why they were excluded. | p. 6 |
| Study characteristics | 17 | Cite each included study and present its characteristics. | Supplementary table 3 |
| Risk of bias in studies | 18 | Present assessments of risk of bias for each included study. | Supplementary table 2 |
| Results of individual studies | 19 | For all outcomes, present, for each study: (a) summary statistics for each group (where appropriate) and (b) an effect estimate and its precision (e.g. confidence/credible interval), ideally using structured tables or plots. | n/a |
| Results of syntheses | 20a | For each synthesis, briefly summarise the characteristics and risk of bias among contributing studies. | Tables 2-4, Supplementary table 4 |
|  | 20b | Present results of all statistical syntheses conducted. If meta-analysis was done, present for each the summary estimate and its precision (e.g. confidence/credible interval) and measures of statistical heterogeneity. If comparing groups, describe the direction of the effect. | n/a |
|  | 20c | Present results of all investigations of possible causes of heterogeneity among study results. | n/a |
|  | 20d | Present results of all sensitivity analyses conducted to assess the robustness of the synthesized results. | n/a |
| Reporting biases | 21 | Present assessments of risk of bias due to missing results (arising from reporting biases) for each synthesis assessed. | n/a |
| Certainty of evidence | 22 | Present assessments of certainty (or confidence) in the body of evidence for each outcome assessed. | pp. 6, 12-14 |
| **DISCUSSION** | | |  |
| Discussion | 23a | Provide a general interpretation of the results in the context of other evidence. | pp. 12-14 |
|  | 23b | Discuss any limitations of the evidence included in the review. | p. 14 |
|  | 23c | Discuss any limitations of the review processes used. | p. 14 |
|  | 23d | Discuss implications of the results for practice, policy, and future research. | pp. 14-15 |
| **OTHER INFORMATION** | | |  |
| Registration and protocol | 24a | Provide registration information for the review, including register name and registration number, or state that the review was not registered. | not registered |
|  | 24b | Indicate where the review protocol can be accessed, or state that a protocol was not prepared. | n/a |
|  | 24c | Describe and explain any amendments to information provided at registration or in the protocol. | n/a |
| Support | 25 | Describe sources of financial or non-financial support for the review, and the role of the funders or sponsors in the review. | p. 15 |
| Competing interests | 26 | Declare any competing interests of review authors. | p. 15 |
| Availability of data, code and other materials | 27 | Report which of the following are publicly available and where they can be found: template data collection forms; data extracted from included studies; data used for all analyses; analytic code; any other materials used in the review. | Supplementary tables 2-4 |

**Supplementary table 2. The consensus of quality appraisal.**

| **Article** | **Q1** | **Q2** | **Q3** | **Q4** | **Q5** | **Q6** | **Q7** | **Q8** | **Q9** | **Q10** | **Q11** | **Q12** | **Criteria** | **Grade** | **Verbal assessment *** | **Strenght of evidence** |
| --- | --- | --- | --- | --- | --- | --- | --- | --- | --- | --- | --- | --- | --- | --- | --- | --- |
| Afsar and Umrani 2020 | Y | Y | Y | Y | Y | N | Y | Y | Y | Y | ? | N | Cross-Sectional | 8/12 | good | 4b |
| Agyare et al. 2019 | Y | Y | N | ? | Y | N | ? | Y | Y | Y | ? | N | Cross-Sectional | 6/12 | fail | - |
| Akkaya 2020 | Y | Y | N | N | Y | Y | Y | Y | Y | N | ? | N | Cross-Sectional | 8/12 | good | 4b |
| Albrecht 2010 | Y | Y | Y | N | Y | N | Y | Y | Y | Y | ? | N | Cross-Sectional | 9/12 | good | 4b |
| Altuntas and Baykal 2010 | Y | Y | Y | N | Y | N | Y | Y | Y | N | ? | Y | Cross-Sectional | 9/12 | good | 4b |
| Anand et al. 2012 | Y | Y | Y | N | Y | N | Y | Y | Y | Y | ? | Y | Cross-Sectional | 10/12 | good | 4b |
| Aydan and Kaya 2018 | Y | Y | Y | N | Y | N | Y | Y | Y | Y | ? | Y | Cross-Sectional | 10/12 | good | 4b |
| Bahrami et al. 2012 | Y | Y | Y | N | Y | N | ? | Y | Y | N | ? | N | Cross-Sectional | 7/12 | creditable | 4b |
| Baker et al. 2016 | Y | Y | Y | N | Y | N | Y | Y | Y | Y | ? | N | Cross-Sectional | 9/12 | good | 4b |
| Basit and Duygulu 2018 | Y | Y | Y | N | Y | Y | Y | Y | Y | N | ? | Y | Cross-Sectional | 10/12 | good | 4b |
| Blackstock et al. 2023 | Y | Y | Y | N | Y | Y | ? | Y | Y | Y | ? | Y | Cross-Sectional | 10/12 | good | 4b |
| Booth et al. 2020 | Y | Y | Y | N | Y | N | Y | Y | Y | Y | ? | Y | Cross-Sectional | 10/12 | good | 4b |
| Coxen et al. 2016 | Y | Y | Y | N | Y | N | Y | Y | Y | Y | ? | Y | Cross-Sectional | 10/12 | good | 4b |
| Cregård and Eriksson 2015 | Y | Y | Y | Y | N | N | N | Y | Y | N | - | - | Qualitative | 6/10 | creditable | 3 |
| Downey et al. 2015 | Y | Y | Y | N | Y | N | Y | Y | Y | N | ? | Y | Cross-Sectional | 9/12 | good | 4b |
| Enwereuzor et al. 2020 | Y | Y | Y | N | Y | N | Y | Y | Y | Y | ? | Y | Cross-Sectional | 10/12 | good | 4b |
| Erkutlu and Chafra 2019 | Y | Y | Y | N | Y | N | Y | Y | Y | Y | ? | Y | Cross-Sectional | 10/12 | good | 4b |
| Farag et al. 2019 | Y | Y | Y | ? | ? | N | Y | Y | Y | N | ? | N | Cross-Sectional | 6/12 | fail | - |
| Gider et al. 2019 | Y | Y | Y | N | Y | N | N | Y | Y | N | ? | N | Cross-Sectional | 7/12 | creditable | 4b |
| Hirvi et al. 2020 | Y | Y | Y | Y | Y | Y | Y | Y | Y | N | - | - | Qualitative | 9/10 | high-quality | 3 |
| Ho and Astakhova 2018 | Y | Y | Y | ? | ? | N | Y | Y | Y | Y | ? | N | Cross-Sectional | 7/12 | creditable | 4b |
| Håvold and Håvold 2019 | Y | Y | Y | N | Y | N | Y | Y | Y | N | ? | Y | Cross-Sectional | 9/12 | good | 4b |
| Hämäläinen et al. 2014 | Y | Y | Y | Y | N | Y | N | Y | Y | Y | - | - | Qualitative | 8/10 | good | 3 |
| Jain 2016 | Y | Y | Y | N | Y | N | Y | Y | Y | N | ? | Y | Cross-Sectional | 9/12 | good | 4b |
| Kumar et al. 2015 | Y | Y | Y | N | Y | Y | N | Y | Y | N | - | - | Case study | 7/10 | good | 2 |
| Leiter et al. 2011 | Y | Y | Y | N | Y | N | Y | Y | Y | N | ? | Y | Controlled | 8/12 | good | 2C |
| Linzer et al. 2019 | Y | Y | Y | Y | Y | Y | Y | Y | Y | Y | ? | N | Cohort | 10/12 | good | 3C |
| Masood and Afsar 2017 | Y | Y | Y | N | Y | Y | Y | Y | Y | N | ? | N | Cross-Sectional | 9/12 | good | 4b |
| Mitcheltree 2021 | Y | Y | Y | Y | Y | Y | N | Y | Y | N | - | - | Case study | 8/10 | good | 4D |
| Olvera et al. 2017 | Y | Y | Y | N | Y | N | Y | Y | Y | N | ? | N | Cross-Sectional | 8/12 | good | 4b |
| Reiter and Tzafrir 2021 | Y | Y | Y | N | Y | N | Y | Y | Y | N | ? | N | Cross-Sectional | 8/12 | good | 4b |
| Roczniewska et al. 2020 | Y | Y | Y | N | Y | N | N | Y | Y | N | ? | Y | Cross-Sectional | 8/12 | good | 4b |
| Salas-Vallina et al. 2021 | Y | Y | Y | N | Y | N | Y | Y | Y | Y | ? | Y | Cross-Sectional | 10/12 | good | 4b |
| Scott et al. 2012 | Y | Y | Y | N | Y | Y | N | Y | Y | N | - | - | Case study | 7/10 | good | 4D |
| Solstad and Petterson 2019 | Y | Y | Y | N | Y | N | N | Y | Y | N | - | - | Case study | 6/10 | creditable | 2 |
| Stander et al. 2015 | Y | Y | Y | N | Y | N | N | Y | Y | Y | ? | N | Cross-Sectional | 8/12 | good | 4b |
| Top et al. 2013 | Y | Y | Y | N | Y | N | Y | Y | Y | Y | ? | N | Cross-Sectional | 9/12 | good | 4b |
| Top et al. 2015 | Y | Y | Y | N | Y | N | Y | Y | Y | N | ? | N | Cross-Sectional | 8/12 | good | 4b |
| Top and Tekingunduz 2018 | Y | Y | Y | N | Y | N | Y | Y | Y | N | ? | N | Cross-Sectional | 8/12 | good | 4b |
| Walker 2013 | Y | Y | Y | N | Y | N | Y | Y | Y | N | ? | N | Cross-Sectional | 8/12 | good | 4b |
| Wong et al. 2010 | Y | Y | Y | N | Y | N | Y | Y | Y | N | ? | Y | Cross-Sectional | 9/12 | good | 4b |
| Ye and King 2016 | Y | Y | Y | N | Y | N | N | Y | Y | Y | ? | Y | Cross-Sectional | 9/12 | good | 4b |
| Yeatts et al. 2016 | Y | Y | Y | N | Y | N | Y | Y | Y | Y | ? | N | Cross-Sectional | 9/12 | good | 4b |
| Yoo et al. 2019 | Y | Y | Y | N | Y | Y | Y | Y | Y | N | ? | N | Cross-Sectional | 9/12 | good | 4b |

Explanation of character:

Y = Yes,

N = No,

? = Unknown.

* The maximum from grades:

86-100 % = high-quality,

65–85 % = good,

51–64 % = creditable,

0–50 % = fail

**Supplementary table 3. Included studies about trust in the leader and trust in the organization.**

| **Author (s), year and country** | **Aim of the study** | **Data / Sample** | **Design, methods & trust perspective** | **Main outcomes** |
| --- | --- | --- | --- | --- |
| Afsar and Umrani 2020. Pakistan. | To examine the mediating effect of trust and moderating effect of thriving between transformational leadership and nurses innovative work behaviour. | Public healthcare hospitals registered nurses (n=362, response rate 75.4%) and their immediate head nurses (n=42, response rate 84%). Total valid questionnaires (n=326). Convenience sampling. | Cross-sectional, correlational study: survey.  Trust in management and peers. | Transformational leadership is positively related to trust in leader (γ=0.673, p<0.01) and trust in leader is positively related to innovative work behaviour of nurses (γ=0.612, p<0.001). Thriving has a significant moderating effect on the relationship between trust in leader and innovative work behaviour (γ=0.152, p<0.01). |
| Akkaya 2020. Turkey. | To investigate the relationship among sub-dimensions by conducting an empirical analysis from the perspective of health organizations’ administrative personnel. | Administrative personnel working in eighteen healthcare organizations (n=156). Response rate 78 %. Random sampling. | Survey.  Organizational trust. | Trust in organization has positive impact on affective organizational commitment (β=.248, p<0.01), on continuance organizational commitment (β=.372, p<0.01), but has no positive impact on normative organizational commitment (β=.163, p>0.05). Trust in supervisors has also positive impact on affective organizational commitment (β=.555, p<0.01), on continuance organizational commitment (β=.504, p<0.01), and on normative organizational commitment (β=.573, p<0.01). |
| Albrecht 2010. Australia. | A preliminary test of the model, which includes three key antecedents of cynicism toward change: change information, involvement with change and trust in senior management. | Data from two large healthcare sector organisations full time employees (n=425 & 325). Response rates 30% & 31%. | Survey.  Trust in management. | Direct but weak influence of change information on cynicism (-0.29, p>0.01). Involvement in change (-0.26, p>0.01) and trust in senior management (-0.25, p>0.01) directly influence cynicism toward change. Information about change (0.44, p>0.01) and involvement in change (0.32, p>0.01) directly influence trust in senior management. |
| Altuntas and Baykal 2010. Turkey. | To determine the levels of nurses’ organizational trust and organizational citizenship and to investigate relationships between the levels of organizational trust and organizational citizenship behaviors. | University, private and Ministry of Health hospitals nurses (n=482). Response rate 53%. A simple random sampling. | Descriptive and explorative design: survey.  Organizational trust (including trust in manager, institution and coworkers). | Nurses trusted in their institutions (SD=1.27) the least and in their managers the most (SD=1.00). On organizational citizenship behaviour nurses obtained the highest score from the conscientiousness dimension (M=6.21±0.78), followed by altruism (M=5.75±0.99), courtesy (M=5.55±1.16), and civic virtue (M= 5.00±1.39) whereas the sportsmanship dimension was close to the average (M=4.40±1.43). There was a positive, weak, but significant relationship between the “trust in manager” (r=.28, p=.000) and “trust in institution” (r=.36, p=.000) dimensions and organizational citizenship level, and a positive, very weak, but significant relationship between the “trust in coworkers” dimension and organizational citizenship level (r=.23, p=.000). |
| Anand, Chhajed and Delfin 2012. USA. | To examine the influence of autonomy in day-to-day work on commitment of frontline employees to continuous improvement. | One group practice employee’s including physicians, nurse practitioners, nurses, health care technicians, and administrative staff (n=317). Response rate 40%. | Survey.  Trust in management. | Trust in leadership has a strong direct effect (β=0.53, p≤0.01) and a strong positive reinforcing effect on the influence of job autonomy (β=0.13, p≤0.01) on commitment to continuous improvement. The hypothesis regarding the main effect of job autonomy on commitment to continuous improvement was moderately supported (β=0.10, p≤0.10). |
| Aydan and Kaya 2018. Turkey. | To reveal the effect of perception of ethical climate by nurses and secretaries and their level of organizational trust on their whistleblowing intention. | Nurses (n=167) and secretaries (n=202) of one university hospital. Response rate 54%. Convenience sampling. | Survey.  Organizational trust. | Explaining the whistleblowing intention, the direct impact of the organizational trust had negative effect (β=-0.13, p≤0.05); while the ethical climate score had the positive effect (β=0.62, p≤0.05). Organizational trust had direct effect on ethical climate (β=0.39, p≤0.05). Organizational trust indirectly by ethical climate impacted on the whistleblowing (β=0.27, p≤0.05). |
| Bahrami, Hasanpour, Rajaeepour, Aghahosseni and Hodhodineghad 2012. Iran. | To investigate the relationship between organizational trust and nurse administrators’ productivity. | Educational health centers of Isfahan university of medical sciences nurse administrators’: head nurse, clinical supervisor, training supervisors, and metrons (n=165). Random sampling. | Descriptive and correlational design: survey.  Organizational trust. | The means of all organizational trust indicators were about average, with loyalty obtaining the highest (M=3.31) but explicitness obtaining the lowest mean at average level (M=3.00). Regarding the seven components of manager’s productivity indicate the highest mean belongs to ability (M=3.87), while the lowest mean, less than average level is related to organizational support (M=2.88). There was a direct and significant relationship between scores of organizational trust and productivity components (β=0.34, p≤0.01). |
| Baker, Mathis, Stites-Doe and Javadian 2016. USA. | To examine the relationship between followers’ self-perceptions of their own follower and leader abilities. | At six sites healthcare organizations workers (n=199). Response rate 40%. | Survey.  Trust in management. | Followers who builds trust are positively related  to behaviors that inspire a shared vision (β=0.163, p=0.046) and insignificantly to behaviors that encourage the hearts of others (β=0.157, p=0.060). Followers courageous communication is positively related to behaviors that inspire a shared vision (β=0.419, p<0.001) and behaviors that encourage the hearts of others (β=0.336, p<0.001). There was no support between followers who identify with their leaders to behaviors that inspire a shared vision (β=-0.042) and behaviors that encourage the hearts of others (β=-0.015). |
| Basit and Duygulu 2018. Turkey. | To investigate nurses’ organisational trust and intention to stay. | State, university and private hospitals nurses (n=265). Response rate 88%. Stratified sampling method. | Descriptive design: survey.  Organisational trust. | The nurses who had worked for 11–15 years at their current institution were found to trust their colleagues more as compared to the others (p=.007).  For nurses who were satisfied with their current institution, the median trust scores on the ‘trust in manager’ sub-dimension were significantly higher than those of nurses who were not satisfied (p=.049). Similarly, the median trust scores of the ‘trust in manager’ sub-dimension for nurses who were planning to continue working at their current institution in the future were significantly higher than those of their counterparts (p=.008). |
| Blackstock, Cummings and Glanfield 2023. Canada. | To test the assumption about new graduate nurses’ perceptions of nursing leaderships’ control over workload contributing to coworker incivility experiences. | 1015 nurses located across Canada with less than three years of work experience (secondary analysis of prior collected data, n = 3906) | Self-reported survey.  Trust in manager. | Trust in immediate supervisor: mean= 3.76 range = 1–5 SD=.96 Cronbach=.57. The correlation between authentic leadership and trust in management (r=.735), were significant at a two-tailed level. For every one unit increase in the perception of trust in management, coworker incivility decreased by .07 (β=−.07, p=.01) and is significant at the 0.01 level, CI [−.12, −.01]. |
| Booth, Shantz, Glomb, Duffy and Stillwell 2020. USA and UK. | To identify individual and contextual conditions that amplify the effects of supervisor undermining. | Sample 1: Direct-care healthcare workers (i.e., nursing, technician, and patient support staff) from USA (n=248). Response rate 22%.  Sample 2: Workers (n=330). Response rate 70%. | Two surveys.  Trust in workplace management. | High Core self-evaluation (CSE)-high trust in management employees experienced the strongest positive relationship between supervisor social undermining and stress appraisals (β=1.15, p<.001). Employees with high CSE and high trust in management have the most exacerbated turnover intentions (β=.56, p<.01), while high-CSE–low-trust, low-CSE–high-trust, and low-CSE–low-trust hadn’t significant effect. High-CSE-high-trust individuals experience strengthened effects of undermining on felt understanding (β=−.71, p=.00). Felt understanding is negatively related to stress appraisals (β=−.14, p<.00) and turnover intentions (β=−.30, p<.00). |
| Coxen, van der Vaart and Stander 2016. South Africa. | To investigate the influence of authentic  leadership on organisational citizenship behaviour, through workplace trust. | Public health care sector organisations employees (n=633). Response rate 32%. | Cross-sectional survey.  Organizational trust. | Authentic leadership was found to be a significant predictor of trust in the organisation (β=0.60, p<0.01), trust in the immediate supervisor (β=0.82, p<0.01) and trust in co-workers (β=0.48, p<0.01). Both trust in the organisation (β=0.28, p<0.01) and trust in co-workers (β=0.22, p<0.05) were significant, positive predictors of organisational citizenship behaviour. Trust in the immediate supervisor (β=−0.02, p=0.81) and authentic leadership were not significant predictors of organisational citizenship behaviour. Authentic leadership had a significant indirect effect on organisational citizenship behaviour through trust in the organisation (β=0.17, p<0.01) and trust in co-workers (β=0.11, p<0.05). |
| Cregård and Eriksson 2015. Sweden. | To explore the dual role of physician-managers through an examination of perceptions of trust and distrust in physician-managers. | Interviews: Hospital physician-managers (n=8) and nurse-managers (n=8).  Six focus groups of two or three nurse-managers and three physician-managers. | Qualitative approach: interviews and focus group discussions.  Trust in management. | Trust in physician-managers is perceived to increase when physicians think physician-managers make decisions that reflect benevolence and integrity according to the medical logic. Trust in physician-managers is perceived to decrease when physicians think physician-managers are unable to balance the demands and duties of their dual role. Physician-managers have difficulty in combining two roles in one position. |
| Downey, van der Werff, Thomas and Plaut 2015. USA. | To investigate the association of diversity practices with employee engagement, a vital ingredient in overall workplace well-being. | Healthcare organization employees (n=4 597). Response rate 49%. | Online “diversity climate assessment” -survey.  Trust climate. | Employees perceptions of the diversity practices in their organization were directly and significantly related to their levels of engagement at work (β=0.32, p<.001). Trust climate mediates partially and significantly the effect of diversity practices (β=0.22, p<.001) on engagement (β=0.06, p<.001). The indirect effect of diversity practices on engagement is statistically significant only at high levels of inclusion (one standard deviation above the mean; β=0.03, p<.001). There is a strong direct relationship between inclusion and trust climate (β=0.734, p<.001). |
| Enwereuzor, Adeyemi and Onyishi 2020. Nigeria. | To investigate the relationship between ethical leadership and safety compliance, with trust in the leader as the mediator. | Hospital staff nurses (n=237). Response rate 89%. A convenience sampling method. | Questionnaires.  Trust in leader. | There was a direct positive relationship between ethical leadership and trust in the leader (β=0.361, p<0.001). Ethical leadership did not have a significant direct relationship with safety compliance (β=0.007, p=0.226). Trust in leader had a direct positive relationship with safety compliance (β=0.023, p<0.001). There was an indirect positive relationship between ethical leadership and safety compliance through trust in the leader (β=0.008). |
| Erkutlu and Chafra 2019. Turkey. | To examine the relationship between a leader’s behavioral integrity and employee acquiescent silence. | University hospitals front-line nurses (n=913). Response rate 70 %. A cluster random-sampling method. | Questionnaires.  Trust in management. | Leader integrity was positively related to employees’ relational identification (β=0.26, p<0.01) and negatively related to acquiescent silence (β=-0.32, p<0.001). There was a significant effect of relational identification on acquiescent silence (β=-0.27, p<0.01) and mediation effect (β=0.07, p=ns; β=-0.24, p<0.01). Conditional indirect effects of employee silence were weaker and significant in the low and middle political skill condition (low-level effect β=0.049, p<0.05; middle level effect β=0.027, p<0.05), but were strong and nonsignificant in the high political skill condition (high-level effect β=0.007, ns). |
| Gider, Akdere and Top 2019. Turkey. | To determine the perception of physicians about organizational trust, employee commitment and job satisfaction and determine the relationships between them. | Training and research hospitals physicians (n=304). Response rate 18%. A convenience sampling. | Cross-sectional study: A questionnaire.  Organizational trust. | There were two significant predictors of job satisfaction: continuance commitment (β=0.255, p=0.035) and organizational trust (β=0.381, p=0.001). Affective commitment (β=0.032, p=0.579) and normative commitment (β=0.005, p=0.941) were not statistically significant predictors of job satisfaction. One significant predictor of commitment was trust (β=0.318, p=0.001). Job satisfaction was not a statistically significant predictor of employee commitment (β=0.069, p=0.249). |
| Hirvi, Laulainen and Taskinen 2020. Finland. | To address the construction of trust in leader member exchange (LMX) relationships as a multidimensional phenomenon and identify the importance of emotional and social (collective) factors in there. | University hospitals healthcare professionals: leaders (n=5) and practical or registered nurses (n=5). | Qualitative: open interviews with thematic analysis.  Trust in management. | There were found two main elements constructing and in the maintenance of LMX trust relations: contextual and core. Contextual elements include work roles and rules, which are expected to fulfill as employment responsibilities and duties state, and collectivity among each others, like giving positive feedback. Core elements include real interaction with communication and presence. Also participation and leaders visibility among work community, while development of mistrust is recognized. |
| Ho and Astakhova 2018. USA. | To test a conceptual model that delineates how and when job passion translates into engagement | Healthcare professionals which were participants of an online course. Time 1 (n=265) response rate 87%. Time 2 (n=232). Nonrandom sampling. | Survey at two times.  Organizational trust. | When obsessively passionate workers trust their organization (β=.27, p<.001), they report greater levels of organizational engagement (because of increased person-organization fit (β=.08, p<.05)), but not because of demands-abilities fit as an alternative mediator when there were trust in coworkers (β=.04, p=.57) nor that trust in supervisor (β=.04, p=.36). In contrast, when these workers trust both their co-workers and supervisor simultaneously, they report greater levels of job engagement (because of increased demands-abilities fit) (β=.10, p<.05), but not directly without trust. |
| Håvold and Håvold 2019. Norway and Finland. | To improve understanding of how different kinds of power influence trust and motivation in hospitals. | Public hospitals nurses, technicians, administrators, senior nurses, senior physicians, head of departments or physicians (n=137). Response rate 35%. | Survey.  Trust in management. | Trust in the manager can be explained positively and significantly by reward power (β=0.337, p=0.000), referent power (β=0.168, p=0.076) and legitimate power (β=0.227, p=0.006), while coercive power influences trust negatively (β=-0.155, p=0.031). Motivation can be explained positively and significantly by expert power (β=0.251, p=0.039), reward power (β=0.334, p=0.000) and trust (β=0.237, p=0.039). Reward power, referent power, legitimate power and coercive power influence motivation indirectly through trust in the manager. There were no direct significant influence to motivation by referent power (β=0.013, p=0.899), legitimate power (β=0.083, p=0.413) and coercive power (β=0.050, p=0.533) and to trust in manager by expert power (β=0.067, p=0.491). |
| Hämäläinen, Tiirinki and Suhonen 2014. Finland. | To deeper the understanding of how first-line manager confirms mutual trust during the process of change in public health care. | Primary health care team managers (n=10). Convenience sampling. | Thematic interviews and content analysis.  Trust in management. | Trust confirmation requires from the first-line manager functional social interaction, employees emotional guidance, profound and critical thinking, self-reflection, peer learning and -support, from the psychological contract’s perspective. It requires also responsibility taking at communal and individual level, and ability and readiness to change, from the work community perspective. Trust through open communication enhances work well-being during the process of change. |
| Jain 2016. Denmark. | To investigate the effect of vertical trust on distributed leadership (DL) and performance as mediated by job satisfaction, and further to observe the role of DL in carrying out the effect of satisfaction on employees’ performance. | Hospital units’ workers, including health professionals, supporting service staff and administrative staff (n=2 217). Response rate 49%. | A longitudinal, cross-disciplinary field study: survey.  Vertical trust. | The relationship of job satisfaction with trust in unit administration (β=0.24, p<0.01) and with trust in department administration (β=0.18, p<0.01) was found to be significant. Job satisfaction had a positive significant impact on DL (β=0.162, p<0.01) and on employees’ performance (β=0.10, p<0.01). Moreover, DL has affected employees’ performance positively (β=0.12, p<0.01). Overall, job satisfaction has mediated the relationship between trust and DL and performance, and DL has mediated the relationship between satisfaction and performance. |
| Kumar, Osborne and Lehmann 2015. Australia. | To investigate the impact of the clinical governance structure. | Country Health South Australia Local Health Network allied health professionals (AHPs). Quantitative: AHPs (n=189), response rate 42 %. Qualitative: AHPs, supervisors and managers (n=12). Purposive sampling. | Mixed methods study: quantitative survey and qualitative focus group interviews.  Trust in management. | Quantitative: Within the normative domain, AHPs recognised the importance of receiving clinical supervision, valued it and considered it necessary to improve the quality of care (mean 15.2). Within the restorative domain, AHPs reported positive findings in levels of trust and rapport with their supervisors (mean 15) and felt supported by their supervisors (mean 14). Within the formative domain, clinical supervision positively affected AHPs delivery of care and improvement in skills (mean 11.4). Qualitative: Enablers resulted in increased awareness, consistent interpretation of policies and procedures and ongoing follow through. Barriers resulted in time to participate and workload by clinical supervisions, and lack of role clarity. |
| Leiter, Laschinger, Day and Oore 2011. Canada. | To examine the effectiveness of an organizational, unit-level CREW intervention aimed at improving social relationships and civility as a means of improving employee and organizational outcomes. | Hospitals nurses, ward clerks, physicians in phase 1 (n=1 173) and phase 2 (n=907). Survey respondents (n=472) response rate 40%. Intervention group participants: phase 1 (n=262) and phase 2 (n=181), control group: phase 1 (n=911) and phase 2 (n=726). | Quasiexperimental: six-month intervention group (n=8 units) and control group (n=33 units).  Trust in management. | For the civility (β=0.12, p<.05), supervisor incivility (β=-0.17, p<.05), respect (β=0.24, p<.05) and cynicism (β=-0.28, p<.05) measures, the intervention units showed a significant improvement from Time 1 to Time 2, whereas the contrast units didn’t improve (β=0.01, 0.02, 0.05, 0.05, p>.05). Improvement in job satisfaction, commitment and trust from Time 1 to Time 2 there were significance for both the intervention (β=0.36, 0.20, 0.16 p<.05) and contrast units (β=0.12, 0.10, 0.10, p<.05), but the size of change was larger for the intervention group. Absences decreased significantly for both the intervention (β=-0.63, p<.05) and contrast groups (β=0.27, p<.05), but the change was stronger for the intervention group. |
| Linzer, Poplau, Prasad, Khullar, Brown, Varkey, Yale, Grossman, Williams and Sinsky 2019. USA. | To examine organizational characteristics associated with trust. Intervention included workflow redesign or chronic disease management programs, to address clinician stress and burnout. | Primary care practices clinicians: general internists, physicians and nurse practitioners (n=165). Intervention (n=17 groups) and control (n=17 groups). Follow-up after 12 to 18 months. | Prospective cohort study, cluster randomized clinical trial.  Organizational trust. | Compared participants with low trust scores, participants with high trust had higher mean (SD) scores on work control (2.49 [0.52] vs 2.18 [0.45]; p<.001), organizational culture variables, including cohesion (3.11 [0.46] vs 2.51 [0.51]; p<.001), emphases on quality vs productivity (3.12 [0.48] vs 2.58 [0.41]; p<.001) and communication (3.39 [0.41] vs 3.01 [0.44]; P<.001), and values alignment (2.61 [0.59] vs 2.12 [0.52]; p<.001). While all outcomes were more favorable in the high-trust group, only mean (SE) satisfaction score was significantly higher in clinicians with high trust compared with those with low trust (3.99 [0.08] vs 3.51 [0.07]; p<.001). Compared with clinicians in whom trust declined or remained low, clinicians with improved or stable high trust reported higher mean (SD) satisfaction (4.01 [0.07] vs 3.43 [0.06]; p<.001) and lower stress (3.21 [0.09] vs 3.53 [0.09]; p=.02) scores and had approximately half the odds of intending to leave (odds ratio, 0.481; p=.04). |
| Masood and Afsar 2017. Pakistan. | To examine the influence of transformational leadership on nurses’ innovative work behavior through the mediating processes or mechanisms of psychological empowerment, knowledge sharing behavior, and intrinsic motivation. | Public sector hospitals nurses (n=587) and doctors who are nursing supervisors (n=164). Response rate of nurses with 43%. | A cross-sectional study: survey.  Trust in leader. | All results were positively significant. Transformational leadership effected on psychological empowerment (β=.73, p<.001) which in turn effected on intrinsic motivation (β=.31, p<.01) and knowledge sharing behavior (β=.19, p<.05). Intrinsic motivation (β=.33, p<.001) and knowledge sharing behavior (β=.56, p<.05) influenced on innovative work behavior. Empowerment role identity interacted with transformational leadership to influence psychological empowerment (β=.25, p<.01). Trust in the leader interacted with knowledge sharing behavior to influence innovative work behavior (β=.33, p<.001). |
| Mitcheltree 2021. Norway. | To investigate how trust mechanisms may enhance innovation speed by reducing employee decisions to perform defensive routines. The study is related to a hospital and its laboratory service. | Phase 1: Workshop participation and meetings, and project documents.  Phase 2: Interviews with key employees (n=5) from four different laboratories. Convenience sampling. | A qualitative investigation involving a case study and semi-structured interviews.  Trust in management. | Important trust mechanisms to enhance innovation speed are managers’ ability to create a space for employee voice and meaning, so that emotional tension and defensive routines won’t generate. Human-centered focus during the innovation implementation process enhances trust, like participation, communicating expectations, availability and timing. |
| Olvera, Llorens, Acosta and Salanova 2017. Spain. | To evaluate the relationship between the transformational leadership perceived by the work team and the team performance perceived by the supervisor, taking into account the mediator role of horizontal trust perceived by the work team. | Healthcare centers workers (n=388) response rate 53% and supervisors (n=54) response rate 71%. Non-probabilistic, convenience sampling. | Cross-sectional study: questionnaire.  Organizational trust (i.e., horizontal trust). | Transformational leadership (at the team level) is positively and significantly related to team performance (evaluated by the supervisor) (β=.36, p<.05) and horizontal trust (β=.68, p<.001). Horizontal trust (at the team level) is significantly related to team performance (evaluated by the supervisor) (β=.43, p<.01). The relationship between transformational leadership (at the team level) and team performance (evaluated by the supervisor) is no longer significant when controlled by horizontal trust (at the team level) (β=.11, p=.59). |
| Reiter and Tzafrir 2021. Israel. | To examine whether collaboration enhances employees’ knowledge and commitments. | Project participants (n=268): senior managers (n=74), employees (n=110) and customers (n=84). Response rate 60%. Snowball sampling. | Questionnaire.  Trust in manager and team colleagues. | There were significant correlations between collaboration and knowledge (r=.484, p< .01) and quality of treatment (r=.365, p<.05). Significantly projects that demonstrated higher levels of treatment quality exceeded the level of total output of the project (r=.859, p<.01). Trust has a high correlation between various variables: collaboration (r=.814, p<.01); output (r=.762, p<.01); and quality of service (r=.711, p<.01). |
| Roczniewska, Richter, Hasson and von Thiele Schwarz 2020. Sweden. | To investigate if social resources can promote sustainable employability over time. | Regional healthcare organization employees (n=269), whose managers participated in a leadership training intervention. Response rate 24%. | Survey.  Vertical trust. | Individuals high (vs. low) in vertical trust rated their job satisfaction higher (γ=0.23, p<0.001), health (γ=0.14, p=0.021) and job performance better (γ=0.28, p<0.001). Units with better teamwork had more healthy (γ=0.32, p=0.046) and productive (γ=0.45, p=0.07) employees. Transformational leadership was a negative predictor of productivity: (γ=−0.26, p=0.09) and further it didn’t significantly predict health (γ=−0.08, p=0.396)**.** While both teamwork (γ=0.16, p=0.28) and transformational leadership (γ=0.12, p=0.18) were positively linked with job satisfaction, these relationships were not statistically significant. |
| Salas-Vallina, Alegre and López-Cabrales 2021. Spain. | To examine the link between well-being-oriented human resource management (WBHRM) and performance, based on the job demands–resource model and social exchange theory. | Large oranizations from diverse sectors (n=8) and their teams (n=212). One sector was health care teams (n=22). Data gathered from  employees (n=1398), response rate 49%. | A quantitative survey.  Trust in leader. | There were significant indirect path from WBHRM to individual performance through happiness at work (HAW) (β=0.15; p<.05), through exhaustion (β=−0.22; p<.05) and from WBHRM to task performance through trust (β=0.24; p<.05). The positive effect of WBHRM on HAW only appeared when it was reinforced by engaging leadership (β=0.18; p<.01). When this did not occur, effect of WBHRM was not significant (β=.02; p>.05). A positive and significant effect of WBHRM and trust was found (β=0.22; p<.05) and negative effect of WBHRM on exhaustion (β=−0.12; p<.05), which became stronger when managers exhibited engaging leadership behavior (β=0.28; p<.01, β=−0.19; p<.05). |
| Scott, Mathews and Gilson 2012. South Africa. | To investigate dynamics surrounding the proposed implementation of staff allocation strategies responding to broader equity-oriented policy mandates. | In-depth interviews with the institutional managers (n=10). Focus group discussions (n=6) with nurses (n=42) and facility managers (n=6). | Exploratory, case study. Multi-method data collection approaches involving in-depth interviews and focus group discussions.  Workplace trust. | Nurses and mid-level managers had similar understanding of equity and for need to implement a strategy based on equitable service delivery. Still there were resistance to reallocation of staff by both staff nurses and mid-level managers. Behind the resistance were lack of workplace trust between mid-level managers and nurse service providers. The lack of trust had several reasons: nurses felt victimized and undervalued by managers, and not been involved in decision-making and overwhelmed by workload. |
| Solstad and Petterson 2020. Norway. | To investigate how professional staff and middle managers perceive their relationships with top managers several years after the hospital merger. | Merged hospital enterprise professionals answered to survey (n=196), response rate 40%. Interviews with clinical middle managers (n=6). | A qualitative case study: survey and interviews.  Trust in management. | Almost half of the staff felt the top management was unvisible, because they didn’t use interactive management practices. Staff perceived lack of possibilities in involvement decision-making. After merger the levels of control increased and middle managers perceived unfair conditions in resource allocation, which implied in signs of mistrust between the professional staff and the top management. Also geographical distance affected to increased mistrust, when middle managers felt that top managers didn’t trust their workings. This kind of top-down management involves little direct communication. |
| Stander, Beer and Stander 2015. South African. | To determine whether the leadership style of authentic leadership could predict optimism, trust in the organisation and work engagement and to establish whether optimism and trust in the organisation could mediate the relationship between AL and work engagement. | Public hospitals and clinics employees (n=633). Convenience sampling. | Cross-sectional study: survey.  Organizational trust. | Authentic leadership (AL) positively predicted optimism (β=0.45; p=0.001) and trust in the organisation (β=0.68; p=0.001), but did not predict work engagement (β=-0.07; p=0.200). Optimism (β=0.51; p=0.001) and trust in the organisation (β=0.39; p=0.001) in turn positively predicted work engagement. Both indirect effects were significant: the relationship from AL to work engagement through optimism (0.23; 95% CI [0.16, 0.30]; p≤0.001) and from AL to work engagement though trust in the organisation (0.27; 95% CI [0.19, 0.34]; p≤0.001). |
| Top, Tarcan, Tekingündüz and Hikmet 2013. Turkey. | To investigate the relationships among employee organizational commitment, organizational trust, job satisfaction and employees’ perceptions of their immediate supervisors’ transformational leadership behaviors. | Public hospitals employees (n=804) response rate 38%. | Cross-sectional study: self-administered questionnaire. Organizational trust. | Articulating a vision (β=0.288; p<0.05), pay (β=0.094; p<0.05), supervision (β=0.172; p<0.05), affective commitment (β=0.109; p<0.05) and normative commitment (β=0.210; p<0.05) had a significant effect on the organizational trust. Three dimensions of transformational leadership and continuance commitment and seven dimensions of job satisfaction didn’t have a significant effect on organizational trust. Organizational trust (β=0.292; p<0.05), contingent rewards (β=0.159; p<0.05) and communication (β=0.075; p<0.05) had a significant effect on the organizational commitment. All dimensions of transformational leadership and seven dimensions of job satisfaction didn’t have a significant effect on organizational commitment. |
| Top, Akdere and Tarcan 2015. Turkey. | To investigate the perceptions of both public servants and private sector employees (outsourcing) on transformational leadership, organizational commitment, organizational trust and job satisfaction. | Public servants and private sector employees (n=804) response rate 38%. | Survey.  Organizational trust. | The two dimensions of job satisfaction – operating procedures (ß=-0.153; p<0.05) and communication (ß=0.120; p<0.05) – as well as organizational trust (ß=0.284; p<0.05) were significant predictors of organizational commitment of public servants, whereas two dimensions of leadership – individualized support (ß=0.457; p<0.05) and fostering the acceptance (ß=-0.297; p<0.05) – as well as two dimensions of job satisfaction – promotion (ß=0.121; p<0.05) and contingent rewards (ß=0.222; p<0.05) – and organizational trust (ß=0.291; p<0.05) were the significant regressors of organizational commitment of private sector employees. Organizational trust has a significant effect on overall organizational commitment as well as its three dimensions for public servants (ß=0.263; p=0.000, ß=0.142; p=0.020, ß=0.217; p=0.000) and private employees (ß=0.301; p=0.000, ß=0.173; p=0.001, ß=0.183; p=0.001). |
| Top and Tekingündüz 2018. Turkey. | To determine the effect of distributive justice, procedural justice, interactional justice, cognition-based trust, and affect-based trust on job stress and explore the relationship among these. | Public hospital organizations health personnel (n=432). Response rate 53 %. | Descriptive, cross-sectional study: survey.  Organizational trust. | Interpersonal justice (β=.234; p<.05) and distributive justice (β=-.125; p<.05) were important predictors of job stress. Organizational trust (β=-.155; p=.099, β=-.053; p=.539) was not an important predictor for job stress. Procedural justice (β=.591; p=.0001) and interactional justice (β=.108; p=.011), but not distributive justice (β=.056; p=.212), were significant predictors in cognition-based trust. Procedural justice was a significant predictor (β=.547; p=.0001) in affect-based trust, while interactional justice (β=.065; p=.160), and distributive justice (β=.047; p=.341) were not significant predictors. |
| Walker 2013. Australia. | To investigate the positive and negative outcomes associated with employer breach and employee fulfillment of the psychological contract of safety. | Hospitals employees, most of them nurses (n=438). Response rate 33%. | Survey.  Organizational trust. | When employee was being injured in the workplace, it was found to lower perceptions of trust in the employer (-.32, p<.01) and increase perceptions of employer breach of safety obligations (.16, p<.05). Trust in the employer significantly influenced perceived employer breach of safety obligations such that lowered trust resulted in higher perceptions of breach (-.71, p<.001). High perceptions of employer breach resulted significantly in low employee fulfillment of obligations (-.29, p<.001). Trust (.29, p<.001) and perceptions of breach (-.64, p<.001) significantly influenced safety attitudes, but not safety behavior. Fulfillment of employee safety obligations significantly impacted safety behavior (.74, p<.001), but not safety attitudes. |
| Wong, Laschinger and Cummings 2010. Canada. | To test a theoretical model linking authentic leadership with staff nurses’ trust in their manager, work engagement, voice behaviour and perceived unit care quality. | Acute care hospitals registered nurses (n=280). Response rate 48 %. Random sampling. | A non-experimental, predictive survey design.  Trust in manager. | Authentic leadership had a significant positive direct (ß=0.43, p<0.001) and an indirect effect on trust through personal identification (ß=0.26, p<0.001) and an indirect effect on work engagement (ß=0.22, p<0.001). Trust had a significant positive direct effect on work engagement (ß=0.19, p<0.001) and in turn, work engagement had a significant direct positive effect on voice (ß=0.22, p<0.001) and perceived quality (ß=0.23, p<0.001). There was no relationship between voice behaviour and quality, such as between social identification and trust. Authentic leadership had significant albeit small positive indirect effects on voice (ß=0.09, p<0.001) and quality (ß=0.12, p<0.001). |
| Ye and King 2016. USA. | To substantiate the trade-off faced by management when adopting a productivity orientation and to investigate trust in management as a means for attenuating the potentially downside effects of adopting such an orientation. | Five different health-care organizations frontline employees: registered nurses (RNs) and licensed practical nurses (LPNs) (n=879). Response rate 24 %. | Questionnaires.  Trust in management. | Productivity orientation was positively associated with frontline employee’s productivity performance (ß=0.28, p<0.01) in both high- and low-trust groups. In the low-trust group, productivity orientation positively associated with employees’ change perceptions (ß=0.14, p<0.05), which were positively associated with role stress (ß=0.45, p<0.01). Further, role stress was negatively associated with employees’ quality performance (ß=-0.23, p<0.01) and job satisfaction (ß=-0.44, p<0.01). In the high-trust group, productivity orientation wasn’t significant with employees’ change perceptions (ß=0.08, p>0.05), which in turn found to be positively associated with role stress (ß=0.45, p<0.01). Further, role stress was found to have a negative relationship with job satisfaction (ß=-0.26, p<0.05), but the relationship with quality performance was non-significant (ß=-0.04, p>0.10). |
| Yeatts, Shen, Yeatts, Solakoglu and Seckin 2016. USA. | To identify factors associated with shared decision-making (SDM) between direct care workers (DCWs) and nurse managers. | DCWs (n=372) working within 11 NHs. Response rate 78 %. | A self-administered instrument.  Trust in management. | Work design and interpersonal relationships between nurse management and DCWs were most important to SDM, within variables of feedback (β=.307; p≤.01), information exchange (β=.237; p≤.01), and management trust (β=.182; p≤.05). Organizational characteristics were also important, including having wages linked to performance (β=.131; p≤.05) and the availability of training (β=.100; p≤.05). |
| Yoo, Zhang and Yun 2019. Korea. | To investigate the effects of explicit and tacit knowledge sharing on clinical decision-making abilities and the mediating role of trust among registered nurses. | Nurses (n=210) in four general hospitals. Random, convenience sampling. | Nonexperimental, structural equation-based modelling study: self-report questionnaires.  Organizational trust. | Findings don’t confirm the direct positive effects from tacit knowledge sharing to clinical decision-making abilities (ß=0.08, p=0.54) and the effects from explicit knowledge sharing to trust (ß=0.24, p=0.19). Instead, findings confirm the direct positive and significant effect from explicit knowledge sharing to clinical decision-making abilities (ß=0.31, p=0.04), as from tacit knowledge sharing to trust (ß=0.48, p<0.01) and from trust to clinical decision-making abilities (ß=2.82, p=0.02). Statistically significant indirect effects are between tacit knowledge sharing with clinical decision-making abilities via trust (ß=0.13, p=0.04). |

**Supplementary table 4. Definitions of trust from the data.**

| Article **and the authors of definition creator** | **Definition** |
| --- | --- |
| Anand et al. 2012 /  **Fleishman and Harris 1962** | Trust in leadership relates to employees’ beliefs in leadership’s concern for the workplace needs of subordinates. |
| Jain 2016 /  **Boon and Holms 1991** | Trust as a state involving confidential positive expectation about another’s motives with respect to oneself in situations entailing risk. |
| Altuntas and Baykal 2010, Stander et al. 2015, Top et al. 2015, Basit and Duygulu 2018, Gider et al. 2019, Blackstock et al. 2023 /  **Cummings and Bromiley 1996** | Organizational trust is: The belief of an individual or a group as a whole that individuals or the organization will make every effort, whether explicit or implied, in good faith to act in accordance with commitments; that honesty in relationships will be ensured as a consequence of commitments; and that involved people will not seek to take advantage of others even if they have such opportunities. |
| Albrecht 2010 /  **Mayer, Davis and Schoorman 1995** | Employee’s “willingness to act on the basis of the words, actions and decisions of senior management under conditions of uncertainty or risk”. |
| Wong et al. 2010, Cregård and Eriksson 2015, Baker et al. 2016, Basit and Duygulu 2018 / **Mayer, Davis and Schoorman 1995** | The willingness of a party to be vulnerable to the actions of another party based on the expectation that the other will perform a particular action important to the trustor, irrespective of the ability to monitor or control that other party. |
| Bahrami et al. 2012, Akkaya 2020 /  **Mayer, Davis and Schoorman 1995** | The organizational trust is referred to the positive expectations of the employees and their expectations about competency, reliability, and benevolence. |
| Top and Tekingunduz 2018, Hirvi et al. 2020, Mitcheltree 2021, Blackstock et al. 2023 /  **McAllister 1995** | Cognition-based trust refers to others’ opinions about a person’s reliability, trustworthiness, honesty, and loyalty. It involves beliefs about one’s competence, capability, and reliability. |
| Top and Tekingunduz 2018, Hirvi et al. 2020, Mitcheltree 2021, Blackstock et al. 2023 /  **McAllister 1995** | Affect-based trust refers to a strong and special relationship that is reflected by the affective bond developing as a result of mutual care and interest between individuals. |
| Downey et al. 2015, Ye and King 2016, Håvold and Håvold 2019, Enwereuzor et al. 2020 / **Rousseau, Sitkin, Burt and Camerer 1998** | A psychological state comprising the intention to accept vulnerability based upon positive expectations of the intentions or behavior of another. |
| Walker 2013, Stander et al. 2015 /  **Gilbert and Tang 1998** | Trust in the organisation can be described as a sense of confidence in and support towards one’s employer, that the employer will fulfill commitments or obligations made to the employee. It is the conviction that an individual holds that the organisation that employs them has their best interest at heart. |
| Afsar and Umrani 2020 /  **Tschannen-Moran and Hoy 2000** | Trust echoes the confidence that an individual has in another’s competence and willingness to work in a fair and ethical manner. |
| Ho and Astakhova 2018 /  **Dirks and Ferrin 2001** | An attitude that is referent-focused, such that one can trust in (i.e. be willing to be vulnerable to) one’s coworkers, supervisors and the organization in general. |
| Solstad and Petterson 2019 /  **Tomkins 2001** | The adoption of a belief by one party in a relationship that the other party will not act against his or her interests. |
| Altuntas and Baykal 2010 /  **Demircan and Ceylan 2003** | The way an employee perceives the support offered by the organization, and his/her confidence in leaders or associates that they are honest and true to their word. |
| Coxen et al. 2016 /  **Ferres 2003** | Workplace trust is conceptualised as the consistency between the perceptions of an individual with regard to a trust referent (organisation, immediate supervisor and co-worker) and the individual’s subsequent actions. |
| Masood and Afsar 2017 / **Gillespie 2003** | Reliance-based trust= a person’s willingness to depend on his/her leader. |
| Masood and Afsar 2017 / **Gillespie 2003** | Disclosure-based trust= a person’s willingness to disclose personal or work-related information to his/her leader. |
| Altuntas and Baykal 2010 /  **Saran, Özgur, Khorshid, Vatan, Yalcınkaya and Demircioglu 2004** | A feeling of confidence and commitment without the perceptions of fear, hesitation and doubt, where the person believes that he/she will receive support and collaboration in resolving problems in times of need without any underlying, ulterior motives and/or negative thoughts on the part of others. |
| Altuntas and Baykal 2010 /  **Yucel 2006** | Expectations of individuals, groups or organizations from individuals, groups or organizations with which they are in mutual interaction that they will make ethical decisions and will develop behaviors that are based on ethical principles. |
| Ho and Astakhova 2018 /  **Schoorman, Mayer and Davis 2007** | Trust is defined as an individual’s willingness to be vulnerable to the actions of another party based on the expectation that the latter will perform a particular action. |
| Top et al. 2013 /  **Tanner 2007** | Organizational trust is defined “as one’s overall faith and confidence in the qualities and abilities of specific others (e.g., coworkers, groups, management) within an organization along with the willingness to depend on them and be vulnerable with them”. |
| Basit and Duygulu 2018 /  **Gill and Sypher 2009** | Organisational trust is the belief or commitment in the honesty, strength, ability, and confidence of the organisation’s members, managers, and institutions. |
| Olvera et al. 2017, Basit and Duygulu 2018 /  **Tan and Lim 2009** | An employee’s willingness to be vulnerable to the actions of the organization, whose behavior and actions he or she cannot control. |
| Afsar and Umrani 2020 /  **Gao, Janssen and Shi 2011** | Trust in the leader has been defined as an employee’s psychological state, which involves positive expectations about the leader’s intentions or behaviours in situations entailing risk. |
